# Use of Double-Layer Carotid Stents Is Associated with Improved Patient Survival and Lower Neurological Complications: A Single-Center Retrospective Observational Study

**DOI:** 10.3390/jcm14030888

**Published:** 2025-01-29

**Authors:** Kipras Mikelis, Marius Kurminas, Givi Lengvenis, Radvilas Jančiauskas, Nerijus Misonis, Povilas Budrys, Rokas Šerpytis, Andrius Berūkštis

**Affiliations:** 1 Department of Radiology, Nuclear Medicine and Medical Physics, Institute of Biomedical Sciences, Faculty of Medicine, Vilnius University, 03101 Vilnius, Lithuania; marius.kurminas@santa.lt (M.K.); givi.lengvenis@santa.lt (G.L.); 2 Faculty of Medicine, Vilnius University, 03101 Vilnius, Lithuania; radvilas.janciauskas@gmail.com; 3 Clinic of Cardiac and Vascular Diseases, Institute of Clinical Medicine, Faculty of Medicine, Vilnius University, 03101 Vilnius, Lithuania; nerijus.misonis@santa.lt (N.M.); rokas.serpytis@santa.lt (R.Š.); andrius.berukstis@santa.lt (A.B.); 4 Interventional Cardiology Centre, Cardiology Clinic, Klaipeda University Hospital, 92288 Klaipeda, Lithuania; povilas.budrys@kulig.lt

**Keywords:** carotid artery stenting (CAS), single-layer stent, double-layer stent, stroke

## Abstract

**Background/Objectives**: Dual-layer stents (DLS) with micromesh technology may offer better protection from plaque protrusion compared to single-layer stents (SLS), but little data are available. The aim of this study is to compare clinical outcomes of elective carotid artery stenting for asymptomatic and symptomatic patients treated for primary CAS with DLS or SLS in a high-volume center. **Methods**: This study is a single-center retrospective cohort study and included patients who underwent elective CAS between December 2006 and September 2023. The final analysis included patient baseline characteristics, postoperative complications and patient outcomes. **Results**: A total of 573 patients underwent elective carotid artery stenting in the study period. Most of the 573 patients undergoing CAS were male (62.5%), and the median age of patients at the time of CAS was 70 years. Of the 573 eligible patients, 43.5% (n = 249) were asymptomatic and 56.4% (n = 323) were symptomatic. Analyzing neurological complications, it was found that the only factor that had a statistically significant effect was the type of stent used. Patients who had a carotid stenting procedure using a single-layer carotid stent had statistically significantly more periprocedural neurological complications (8.3% (n = 35)) than the double-mesh stent group (2% (n = 3)), mostly due to more transient ischemic attacks in the single-layer stent group (4% (n = 17)) compared to the double-mesh group (0.7% (n = 1)). **Conclusions**: The use of carotid double-layer stents is associated with a low rate of periprocedural and postprocedural events.

## 1. Introduction

Stroke is the most common serious manifestation of cerebrovascular disease and the leading cause of hospitalization for neurologic disease [1]. The reason why it poses a significant burden on healthcare is the fact that it is the second leading cause of both disability and death worldwide, exceeded only by ischemic heart disease [2,3]. In developed countries, stroke is the primary cause of disability and the third most common cause of mortality [4]. The high-cost burden arises not only for the initial management of the disease but also affects the long-term period of rehabilitation, community care costs and loss of earnings [3].

An internal carotid artery that is narrowed by 50% or more by atherosclerotic plaques is found in 15–20% of patients who present with ischemic stroke or transient ischemic attack [5]. Stenosis of internal carotid arteries caused by atherosclerosis is the underlying cause of 8–15% of ischemic strokes and is called symptomatic carotid stenosis. One to two percent of the adult population have asymptomatic carotid stenosis [4].

Examination of carotid arteries by doppler ultrasound is the main non-invasive imaging method for assessing the degree of stenosis of internal carotid arteries as well as in candidate selection for surgical or endovascular procedures [4]. Moderate (50–79%) and severe (80–99%) stenosis of carotid arteries is a crucial public health issue affecting ~10% of the general population by their 8th decade [6]. A high prevalence of vascular risk factors such as hypertension, diabetes, hypercholesterolemia, and cigarette smoking in an aging population likely explains this high disease burden [7]. Atherosclerotic plaques can rupture and cause thrombosis and emboli, which can lead to stroke if they occur in a carotid artery [8].

Risk of stroke and stroke-related morbidity and mortality can be decreased by treating the stenosis; there are two treatment methods available: carotid artery endarterectomy (CEA) and carotid artery stenting (CAS) [9]. Carotid artery stenting is the standard treatment method of carotid artery stenosis. It is a less invasive method that can be used under mild sedation, requires no surgical incisions, has no risk of cranial nerve palsy and has less cardiovascular complications [4]. Intervention in patients with symptomatic carotid artery stenosis that is higher or equal to 50% was found to be highly beneficial, provided that the complication rates were low. As a method of interventional treatment for carotid stenosis, CAS has been studied and compared with CEA and showed similar results [9,10,11]. Over the last 30 years, CAS frequency of use has been increasing, and clinical outcomes are improving with advancements of technology. Innovative endovascular techniques have created a quickly evolving field of medical devices which allow for safe and minimally invasive alternatives to carotid endarterectomy for carotid revascularization [12,13,14]. This procedure with dedicated equipment, including devices for proximal or distal embolic occlusion, has minimized procedural strokes [15]. Crucial patient characteristics such as age, relevant vascular anatomy and comorbidities should be considered before performing any carotid artery stenting procedure [16]. Operator experience gained over the years, dual-layer stents and other new technologies with enhanced embolic protection continue to improve the outcomes of procedures. Of particular interest remains the new generation double-layer stent design, which not only improves the stents’ adaptability to the complex anatomy of the carotid arteries due to its inner layer that offers radial strength and a flexible outer layer that accommodates to vessel wall movement, but also improves plaque coverage and prevents embolic complications by forming a double-mesh barrier that shields from potential debris [17,18].

Carotid artery stenting has been performed in our center since 2006. During more than 15 years of performing this procedure, the materials used to treat carotid artery stenosis have improved, as did our operator experience, and this procedure has become a standard of treatment for both symptomatic and asymptomatic carotid stenosis.

The purpose of this study is to analyze the efficacy and safety of elective carotid stenting in our center and identify the clinical and technical factors that influenced the outcomes of these procedures.

## 2. Materials and Methods

The study protocol was approved by our regional ethics review board (Permission number 158200-18/3-1018-515). A single-center retrospective cohort study was performed. Patients who underwent an elective carotid artery stenting procedure due to symptomatic and asymptomatic carotid artery stenosis between December 2006 and September 2023 were included in the study. We excluded any cases of carotid stenting during stroke tandem occlusion or acute carotid occlusion stenting. Since endarterectomies are rarely performed in our center, most patients were selected to perform a stenting procedure; however, in a few cases where patients’ anatomy was deemed too risky for an endovascular procedure or patients chose to have a surgical treatment, endarterectomy was performed, and these patients were excluded from the study.

All patients were prepared for the planned procedure by loading with double antiplatelet therapy. Stenting procedures were performed under local anaesthesia. All procedures were performed by four endovascular specialists who are experienced in neurointerventional radiology. The carotid stenosis grade was measured before the procedure using computed tomography angiography (CTA) or duplex ultrasound; however, the final recorded stenosis grade was measured during angiography using North American Symptomatic Trial Collaborators (NASCET) criteria.

We recorded patients’ demographic data, concomitant illness, laboratory test values, stenosis grade, procedural parameters and materials used, periprocedural complications and mortality after the procedure.

All patients’ data were anonymized and analyzed using Microsoft Excel 2021 (Microsoft, Seattle, WA, USA) and IBM SPSS Statistics for Windows, version 27 (IBM Corp., Armonk, NY, USA). We looked for statistical association between periprocedural complications and mortality and patients’ concomitant disease, lab values, carotid stenosis and procedural parameters. Pearson’s 2 test was used to test for an association between categorical data and an independent-samples *t*-test for continuous variables. Patients’ comorbidities and demographic variables were adjusted for when testing for associations with complications and mortality after the procedure. The significance level was set at 0.05. All statistical analysis was performed by a professional medical statistician in Vilnius University Faculty of Medicine.

## 3. Results

A total of 573 patients underwent elective carotid artery stenting in the study period. Most of the 573 patients undergoing CAS were male (62.5%), and the median age of patients at the time of CAS was 70 years. Among patients treated with CAS, the most common medical comorbidity was primary arterial hypertension (88.3%), followed by dyslipidaemia (65.1%), coronary heart disease (49%) and peripheral arterial disease (27.1%) (Table 1).

Of the 573 eligible patients, 43.5% (n = 249) were asymptomatic and 56.4% (n = 323) were symptomatic. The most common presentation of symptomatic stenosis was stroke 58.5% (n = 189), followed by transitory ischemic attack 41.5% (n = 134) (Table 2).

A total of 49% (n = 281) of the patients had a stenting procedure performed in the right internal carotid artery (ICA) and 51% (n = 292) of patients in the left ICA (Table 3).

**Figure 1:**
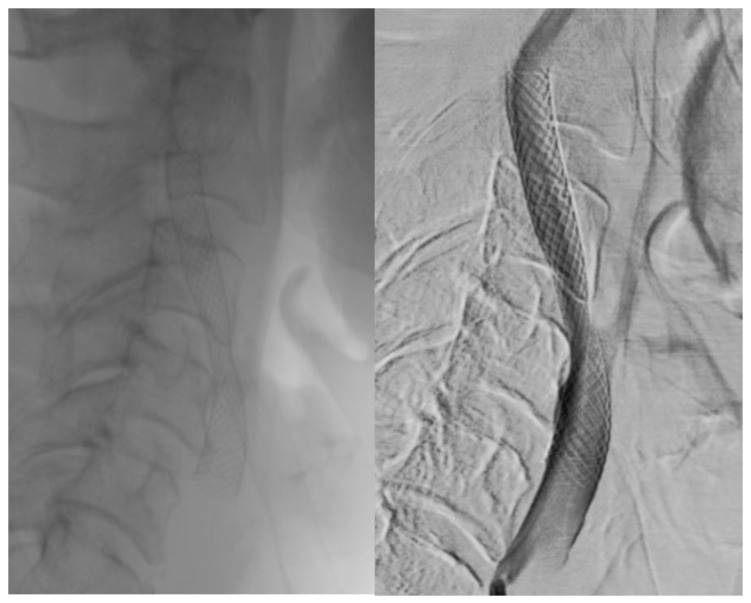
Single-layer micromesh stent (Wallstent-Boston Scientific).

The mean stenosis grade in stented internal carotid arteries and the stenosis grade of contralateral side ICA and common carotid as well as vertebral arteries are shown in Figure 1.

Femoral access was used in 93.4% (n = 535) of the cases, and in 6.6% (n = 38) of the cases, radial or brachial access was used (mostly due to type III aortic arch or complicated groin access).

The median procedure time was 45 min (ranging from 15 to 185 min). All our procedures used a distal protection device. Most of the time 96% (n = 550), one stent was sufficient to cover the lesion; however, in some cases 3.8% (n = 22), two carotid stents were used, and in one case (0.2%), three stents were used (Table 4).

During the years of performing this procedure, several types of carotid stents were used. The most common single-layer stent used was Carotid WALLSTENT™ Monorail Endoprosthesis-Boston Scientific (Boston, MA, USA) 38.0% (n = 218), followed by Abbott XACT-Abbott Vascular Devices (Galway, Ireland) 21.8% (n = 125) and Protégé™ Peripheral Stent System-Medtronic Operational Headquarters (Minneapolis, MN, USA) 14.0% (n = 80) (Table 5 and Table 6).

After 2015, there was an increased usage of new types of double-layer carotid stents: Roadsaver™ Carotid Artery Stent (Terumo Interventional Systems) 23.0% (n = 132) and CGuard carotid stent system (Inspire MD, Boston, MA, USA) 3.1% (n = 18) (Table 7).

**Figure 2:**
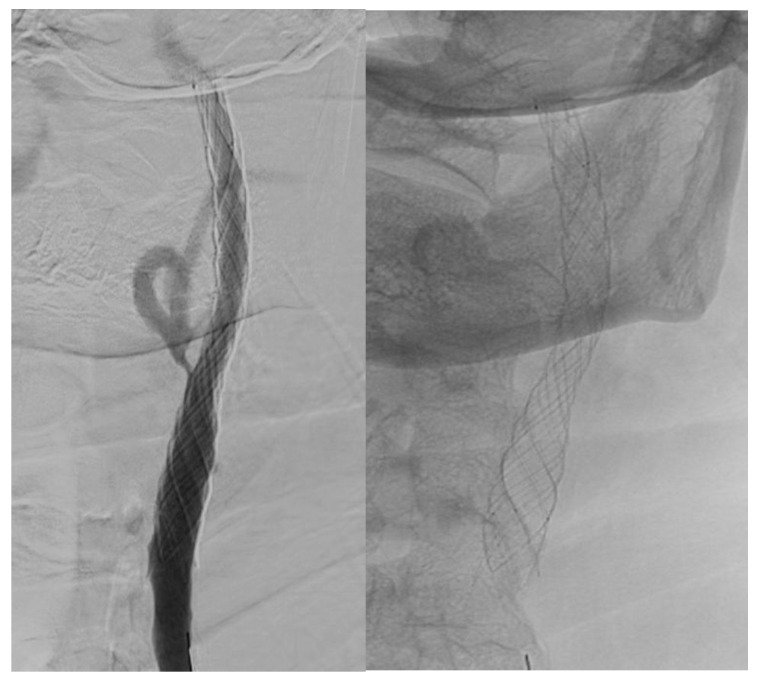
Double-layer micromesh stent (Roadsaver-Terumo Interventional Systems).

**Figure 3:**
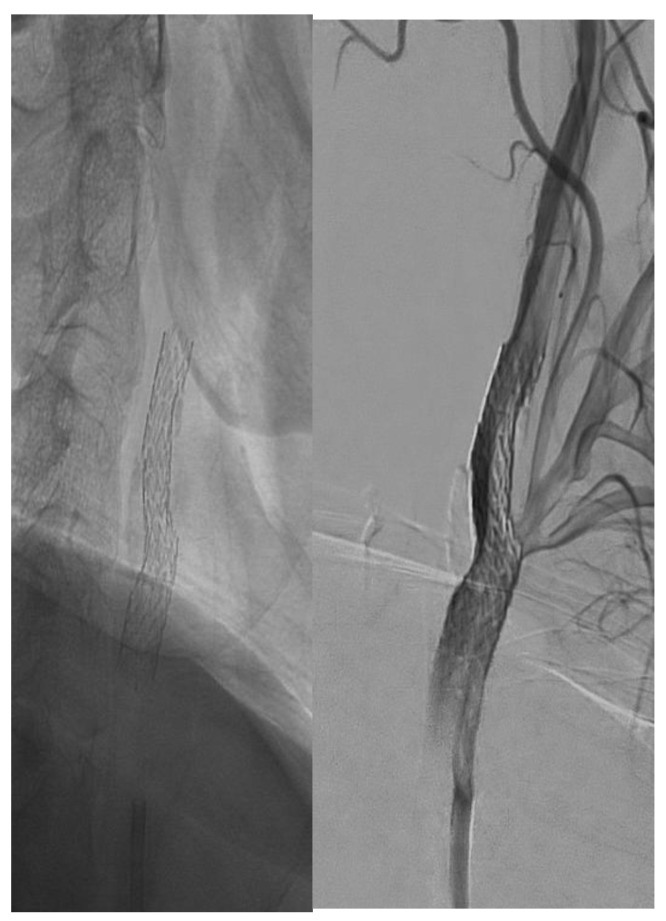
Double-layer micromesh stent (Cguard-Inspire MD).

**Figure 4:**
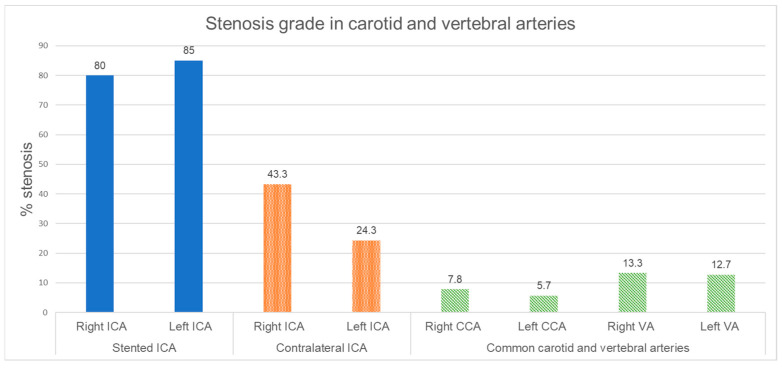
Stenosis grade in carotid and vertebral arteries. Internal carotid artery: ICA, common carotid artery: CCA, vertebral artery: VA.

Visual differences between single- and double-layer carotid stents as seen during the angiography procedure are illustrated in Figure 2, Figure 3 and Figure 4.

Overall, 73.8% (n = 423) single-layer stents and 26.2% (n = 150) double-layer stents were used. The median length of stents was 40 mm (range 8–80 mm), whereas the median width of the stent was 7 mm (range 3.5–11 mm) (Table 5, Table 6 and Table 7).

There were 8.9% (n = 51) of patients who had periprocedural complications. Neurological complications consisted of stroke 3.4% (n = 20) and TIA 3.1% (n = 18); 2.2% (n = 13) of the specimens had other complications (access-site complications, hematomas (10 (1.6%)) and pseudoaneurysms (3 (0.5%)), treated conservatively). During the periprocedural period, 1.9% (n = 11) of patients died (Table 8).

**Figure 5:**
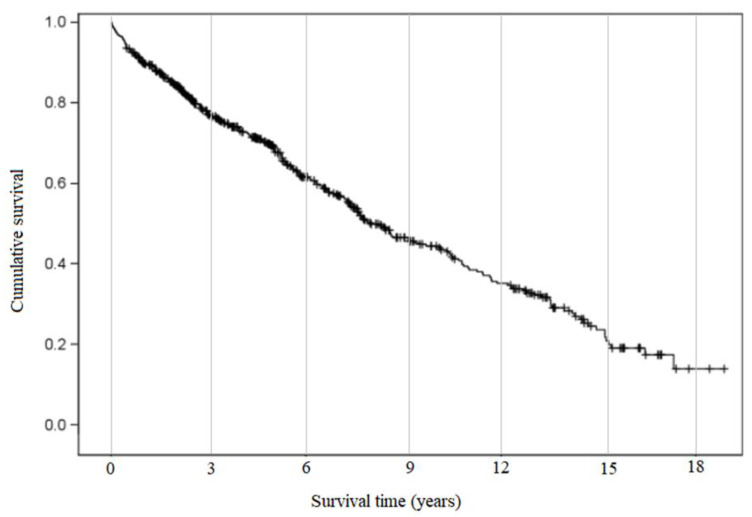
Median overall survival after carotid stenting procedure.

The median overall survival after the carotid stenting procedure was 7.29 years, (confidence interval 6.71 to 8.67 years), and one-month survival was 98.6% (Figure 5).

We looked for statistically significant differences in demographic and procedural factors between the group of patients who developed complications after the procedure and those who did not. Patients who developed complications were older (72.45 vs. 69.9 years (*p* = 0.05)), and their procedure time was longer (57.92 vs. 48.85 min (*p* = 0.01)). Concomitant illnesses such as hypertension were more common in the group that developed complications. When examining other procedural factors, a significantly smaller complication rate was found in the double-mesh stent group (4.7% (n = 7) vs. 10.4% (n = 44) (*p* = 0.03)) (Table 9).

Furthermore, when analyzing just neurological complications like periprocedural stroke and transient ischemic attacks, we found that the only factor that had a statistically significant effect on these complications was the type of stent used. Patients who had a carotid stenting procedure using a single-layer carotid stent had statistically significantly more periprocedural neurological complications (8.3% (n = 35)) than the double-mesh stent group (2% (n = 3)), mostly due to more transient ischemic attacks in the single-layer stent group (4% (n = 17)) compared to the double-mesh group (0.7% (n = 1)). Strokes were also more common in the single-layer group—4.3% (n = 18) vs. 1.3% (n= 2); however, this difference was not statistically significant (Table 10).

Similar trends were also seen when looking into mortality after the stenting procedure; however, not statistically significant, periprocedural mortality was more often seen after stenting using a single-layer carotid stent—2.5% (n = 10), whereas only one patient died in the periprocedural period in the double-mesh stent group (0.7%).

**Figure 6:**
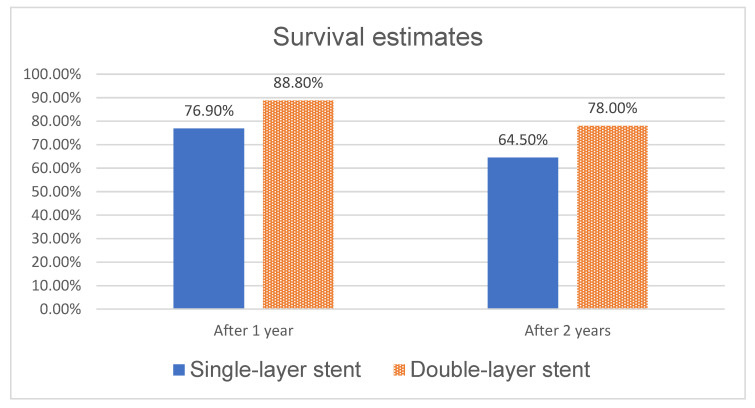
Survival after stenting procedure—different stent types.

**Figure 7:**
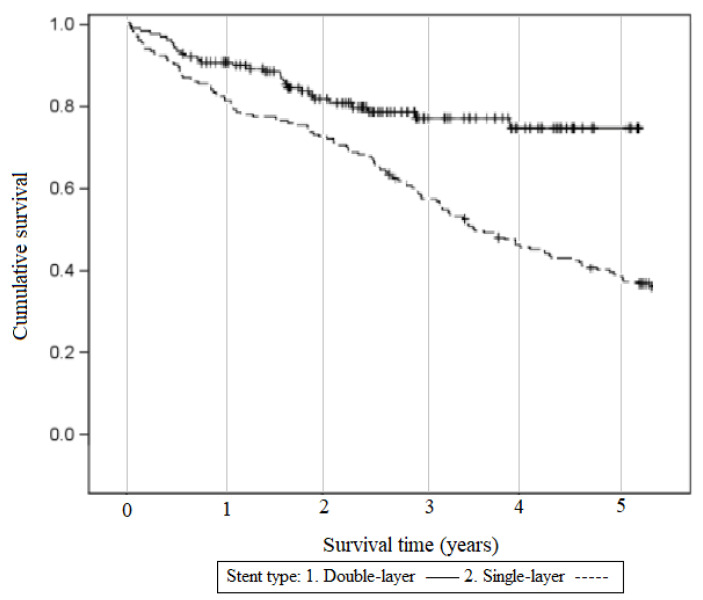
Survival after stenting procedure—different stent types.

We also looked at overall survival after the stenting procedures. Differences between such groups as symptomatic or asymptomatic stenosis were slight and not statistically significant; however, once more, a significant difference was seen between survival after stenting with a double-mesh stent and a single-mesh stent. Better survival was seen in the double-mesh group after one month, one year, two years and overall, as illustrated in Figure 6 and Figure 7 (*p* < 0.01).

## 4. Discussion

Preventing stroke is the primary goal in the treatment of carotid artery stenosis. CAS is one of the main procedures that is considered for treating severe (more than 50%) stenosis caused by atherosclerotic plaque in the carotid artery lumen [19].

The safety of carotid stenting procedures was shown in many studies. A 2022 meta-analysis of the main randomized control trials comparing carotid artery stenting and carotid endarterectomy showed that the main outcomes (stroke, death and myocardial infarction) were present in 3.5% of cases in the 30 days following the stenting procedure. Our rates of periprocedural stroke and death are higher but comparable to the results found in the literature (cumulative periprocedural stroke and mortality rate—5.3%). Although our rates of postprocedural stroke are like those found in this review (3.4% vs. 3.0%), the larger incidence of periprocedural mortality could be attributed to the advanced age of our patient population as well as a large percent of cardiovascular comorbidities [20].

Common risk factors for complications after the carotid stenting procedure are symptomatic stenosis, advanced age and comorbidities such as hypertension and coronary heart disease [21]. In our analysis, older patients with hypertension, coronary heart disease, as well as a history of myocardial infarction or congestive heart failure, were more likely to have some sort of complication after our procedure. It is also known that risk of periprocedural stroke is respectively lower if a distal embolization protection device is used, which is why these devices were used in all our cases, per protocol. It is important to note that in all our experience, we had no periprocedural myocardial infarctions.

Some of the complications in our cohort were puncture-site-related, a common sequela after any endovascular intervention, and were treated conservatively. When taking only neurological complications—periprocedural stroke and transient ischemic attacks—into consideration, the only statistically significant factor was the type of stent used.

Stent design improvements, continuously developing technical skills, experienced interventionalists and proper patient selection before intervention are what make CAS a safe and effective symptomatic and asymptomatic carotid artery treatment method [22]. A new family of double-layer micromesh-covered stents was introduced in 2013. The architecture of open-cell stents, which provides the best flexibility and apposition with closed cells of micromesh, enabled restoration of the full anatomical vessel structure and limited plaque protrusion, which, in hand with a distal embolization protection system, limited the risk of embolic complications [22,23].

Multiple studies looking at patients who underwent CAS with the implantation of a dual-layer micromesh-covered stent (Roadsaver or CGuard) confirmed a very low rate of complications, around 1%. [22,24,25] Nerla et al.’s retrospective analysis reports that CAS procedures using double-layer stents, such as the Roadsaver, have no cerebrovascular events described at the 30-day follow-up. It appears that patients with lipid-rich plaques are one of the most suitable candidates to receive double-layer stents [25].

Pini et al. published a meta-analysis that analyzed the 30-day stroke rate of 14 selected studies on CAS using double-layered stents. Overall, the stroke rate was only 1.5% among asymptomatic participants. For symptomatic patients, the stroke rate was as low as 1.9%. Tigkiropoulos et al.’s meta-analysis demonstrated that a rate of neurologic events in the CGuard double-layer stent group was only 1%. The mortality of patients who underwent CAS with double-layer stents in this example was 2.3% at the 1-year period [26].

Our experience with double-mesh stents, although obtained in a non-randomized series of real-world patients, seems to be in line with results found in other authors’ research. Use of a double-mesh stent was the only statistically significant factor that influenced periprocedural neurological complications—only two percent of patients who had this new generation of stent implanted had a periprocedural transient ischemic attack or periprocedural stroke (stroke rate—1.3%). The rates of periprocedural mortality in the double-layer stent group were also comparable to the ones found in the literature, as low as 0.7 percent. Even though there is still a lack of large prospective studies comparing single-layer stents with newer ones [27], the findings lead us to conclude that a switch to double-layer stent systems is a reliable way to reduce periprocedural complications commonly associated with the carotid stenting procedure. Moreover, profound patient selection was mentioned to be one of the most important determining factors of subsequent long-term survival. Additionally, a strong association between congestive heart failure and mortality during follow-up was observed [18,28,29]. Although significant, these findings were obtained from a single-center retrospective study, as such, a need for further research prospectively comparing double-layered and single-layered stents is required to have a definitive answer about one stent design being superior to another regarding complications and patient survival and, importantly, long-term patency of different types of stents.

## 5. Limitations

Our study is limited by several factors, first of which is the single-center retrospective design. Furthermore, as Vilnius University Hospital Santaros Klinikos is a tertiary referral center, it is possible that patients underwent management of complications at outside institutions. Thus, the reliability of some results hinges on the precise documentation of comorbidities and the occurrence of complications throughout the follow-up period. This is the same reason why we did not have reliable data on restenosis rates after the procedure since most patients underwent follow-up ultrasound scans at their primary care centers or were lost to follow-up altogether. The lack of reliable follow-up duplex and clinical data was very disappointing since accurate data on restenosis rates would complete the full picture of long-term outcomes of carotid artery stenting in our center, as well as give some insight into differences in restenosis rates of single-layer and dual-layer stents. Moreover, the number of endarterectomies performed at our center was not enough to perform a complete comparative analysis to carotid artery stenting. Such referral bias could also influence complication and mortality rates of our series, since despite anatomical or plaque related characteristics, all patients were referred to CAS. The cause of death was determined by thorough review of the medical records in a national registry and categorized in each case with a reasonable degree of certainty. However, it is possible that the attributed cause of death was incorrect in certain cases because of incomplete or absent documentation. Finally, another factor that was not taken into consideration was the increase in operator experience. A newer generation of stents was only introduced in our center in 2015, as such, the lower complication rate could also be attributed to more years of performing this procedure.

## 6. Conclusions

To conclude, in our experience, carotid artery stenting is a safe and effective procedure to treat both symptomatic and asymptomatic carotid artery stenosis. Proper patient selection, preparation and experienced operators ensure a high success rate, and the use of double-layer stents in combination with a distal protection device can reduce the risk of periprocedural stroke and increase the short-term and long-term patient survival after this procedure. Such technical improvements are an important stepping stone toward shifting the standard of care toward a more minimally invasive approach.

## Figures and Tables

**Table 1:** Demographic data.

Patient’s Characteristics	
Male, n (%)	418 (62.5%)
Female, n (%)	155 (23.2%)
Age, median years (range)	70 (min 45 max 93)
Dyslipidemia, n (%)	373 (65.1%)
Hypertension, n (%)	506 (88.3%)
Coronary heart disease, n (%)	280 (49%)
Peripheral arterial disease, n (%)	155 (27.1%)

**Table 2:** Symptomatic and asymptomatic carotid artery stenosis.

Presentation	
Asymptomatic stenosis, n (%)	249 (43.5%)
Symptomatic stenosis, n (%)	323 (56.4%)
Stroke, n (%)	189 (58.5%)
Transitory ischemic attack, n (%)	134 (41.5%)

**Table 3:** Stenting side.

Side	
Right ICA, n (%)	281 (49%)
Left ICA, n (%)	292 (51%)

**Table 4:** Procedural parameters.

Patient’s Characteristics	
Femoral, n (%)	535 (93.4%)
Radial/Brachial access, n (%)	38 (6.6%)
Procedure time: median and range	45 min (from 15 to 185 min)
The number of stents used	
One, n (%)	550 (96%)
Two, n (%)	22 (3.8%)
Three, n (%)	1 (0.2%)

**Table 5:** Types of stents (overall).

Types of Stents	
Single-layer stent, n (%)	423 (73.8%)
Double layer, n (%)	150 (26.2%)
Median stent length, mm (range)	40 (8–80)
Median stent width, mm (range)	7 (3.5–11)

**Table 6:** Types of stents (single layer).

Stent Type	
Carotid WALLSTENT™ Monorail Endoprosthesis-Boston Scientific (Boston, MA, USA)	218 (38.0%)
Abbott XACT-Abbott Vascular Devices (Galway, Ireland)	125 (21.8%)
Protégé™ GPS Self-Expanding Peripheral Stent System-Medtronic Operational Headquarters (Minneapolis, MN, USA)	80 (14.0%)

**Table 7:** Types of stents (double layer).

Stent Type	
Roadsaver™ Carotid Artery Stent-Terumo Interventional Systems (Tokyo, Japan)	132 (23.0%)
CGuard carotid stent system-Inspire MD (Boston, MA, USA)	18 (3.1%)

**Table 8:** Periprocedural complications.

Complications	
Periprocedural complications, n (%)	51 (8.9)
Neurological complications, n (%)	38 (6.6)
○ Stroke, n (%)	20 (3.4)
○ TIA, n (%)	18 (3.1)
Other complications, n (%)	13 (2.2)
Periprocedural mortality, n (%)	11 (1.9)

**Table 9:** Complication rates and factors.

Factor	Complications	No Complications	*p* Value
Age, years	72.45	69.9	0.05
Procedure time, minutes	57.92	48.85	0.01
Hypertension, n (%)	51 (10.1)	0	<0.01
Coronary heart disease, n (%)	32 (11.4)	19 (6.5)	0.04
Myocardial infarction, n (%)	23 (13.1)	28 (7.1)	0.02
Congestive heart failure, n (%)	25 (12.6)	26 (7.0)	0.02
Double-layer stent, n (%)	7 (4.7)	44 (10.4)	0.03

**Table 10:** Outcomes.

Outcome	SLS	DLS	*p* Value
Neurological complications, n (%)	35 (8.3)	3 (2)	<0.01
Transient ischemic attacks, n (%)	17 (4.0)	1 (0.7)	0.05
Stroke, n (%)	18 (4.3)	2 (1.3)	0.09
Periprocedural mortality, n (%)	10 (2.5)	1 (0.7)	0.18

## Data Availability

All data generated or analyzed during this study are included in this published article.
